# Association Between Serum Aldehydes and Hypertension in Adults: A Cross-Sectional Analysis of the National Health and Nutrition Examination Survey

**DOI:** 10.3389/fcvm.2022.813244

**Published:** 2022-03-07

**Authors:** Yongjian Zhu, Mingjing Liu, Wanrong Fu, Yacong Bo

**Affiliations:** ^1^Department of Cardiology, The First Affiliated Hospital of Zhengzhou University, Zhengzhou, China; ^2^Department of Clinical Medicine, Sanquan College of Xinxiang Medical University, Xinxiang, China; ^3^Department of Respiratory and Critical Care Medicine, The First Affiliated Hospital of Zhengzhou University, Zhengzhou, China

**Keywords:** aldehydes, hexanaldehyde, hypertension, adults, cross-sectional

## Abstract

**Background:**

Exposure to ambient pollutants and chemicals were found to be associated with increased risk of hypertension. However, the relationship between the increased aldehyde exposure and hypertension are still unclear. This study aimed to investigate the potential associations of serum aldehydes levels with prevalent hypertension.

**Methods:**

A total of 1,733 U.S. adults with data on hypertension outcome and serum aldehydes measurement from the National Health and Nutrition Examination Survey 2013–2014 were included. The serum levels of aldehydes were measured *via* an automated analytical method using solid phase microextraction gas chromatography and high-resolution mass spectrometry. Multivariate logistic regression models were adopted to assess the associations between six selected aldehydes exposure (benzaldehyde, butyraldehyde, heptanaldehyde, hexanaldehyde, isopentanaldehyde, and propanaldehyde) and prevalence of hypertension.

**Results:**

The mean age was 48.0 ± 16.7 years and an approximately equivalent of sex distribution was observed (female 49.9%). There seems to be a numerically higher level of hexanaldehyde in participants with hypertension when compared to participants without hypertension (2.6 ± 3.9 ng/mL vs. 2.3 ± 1.1 ng/mL). After adjusting for potential confounders, the odds ratio (OR) for hypertension was 2.15 [95% confidence interval (CI): 1.33–3.51] in participants from the highest quartile of serum hexanaldehyde concentration in comparison to those from the lowest quartile. Subgroup analyses and sensitivity analyses showed generally similar results.

**Conclusion:**

In summary, current evidence suggested that increased serum hexanaldehyde level was positively associated with prevalent hypertension in U.S. adults.

## Introduction

Hypertension is known to affect approximately one third of the world's adult population (1). As one of the most important risk factors for cardiovascular diseases and all-cause mortality (2), an estimated more than 7 million annual deaths are attributed to hypertension (3, 4). Epidemiological studies suggested that both genetic and environmental factors contribute to the development of this disorder. Cumulative evidences demonstrated that lifestyle interventions on modifiable risk factors of hypertension including obesity, dietary intakes, smoking, alcohol drinking, and physical activity, have been recognized as effective methods to reduce the morbidity and mortality (5–10). In recent years, exposure to certain ambient pollutants and chemicals were also found to be associated with increased risk of hypertension (11, 12).

Aldehydes are electrophilic organic compounds which include a number of particular components (13). The natural sources of aldehydes are various. Apart from aldehydes derived from environmental or occupational exposure, aldehydes are also generated by food heating and endogenous enzyme-dependent oxidation (14). In human, biogenic aldehydes are mainly from the peroxidation of sugars and lipids. In the past, the adverse health effects of aldehydes exposure and established diseases have been preliminarily investigated. Accumulation of aldehydes in the body aggregates the oxidative stress and covalent modification of protein, and were involved in the pathophysiology of aging related conditions including tumors, chronic liver and kidney diseases, and Alzheimer's disease (14–16). Under high levels of lipid and glucose oxidation, the cardiovascular systems are susceptible to the effects of endogenous aldehydes (17). Acrolein exposure has been shown to promote the development of systemic dyslipidemia, atherosclerosis and thrombosis (18). Serum acrolein and isopentanaldehyde levels were also demostrated to be positively associated with increased risk of cardiovascular diseases (19–21). However, the relationship between the serum levels of aldehydes and risk of hypertension is still unclear.

Therefore, based on data from the National Health and Nutrition Examination Survey (NHANES), this study was conducted to investigate the potential associations of serum aldehydes levels with prevalent hypertension in the U.S. adults.

## Methods

### Study Population

**Figure 1:**
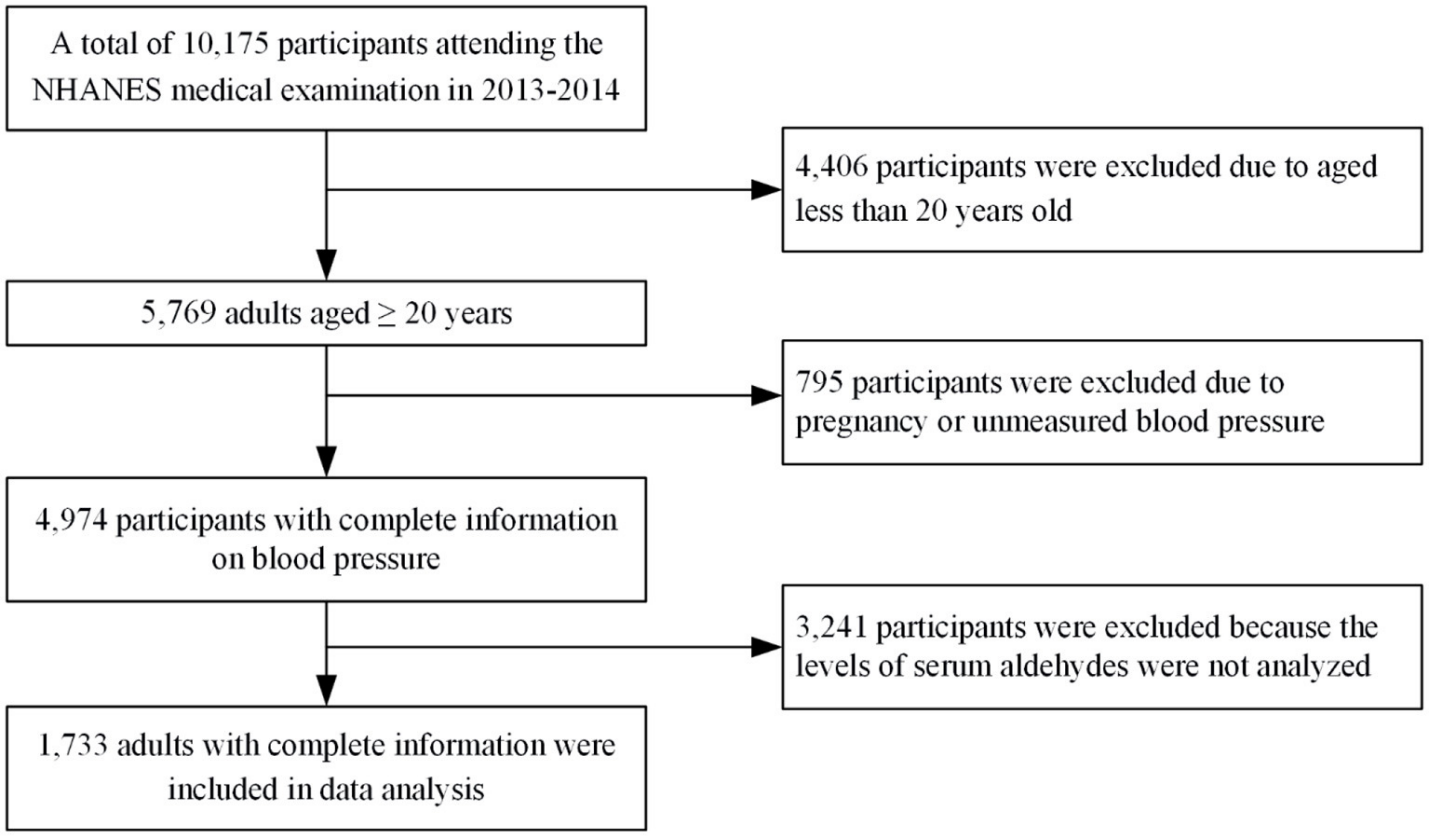
Flowchart of participant selection.

NHANES has been conducted continuously since 1999 by the National Center for Health Statistics (NCHS) at the Centers for Disease Control and Prevention (CDC), which originated from the National Health Survey Act of 1956. This program aims to provide statistical information on the health and nutritional status of representative population in the U.S. by the means of a multistage, probability sampling survey^1^. Data collecting was performed using household interviews, self-reported questionnaire, physical examinations, and laboratory test. The NHANES data could be publicly accessed from the internet (https://www.cdc.gov/nchs/nhanes/index.htm). In this study, we initially included participants enrolled in 2013–2014 when aldehydes measurements were available (*n* = 10,175). Among them, 4,406 individuals aged <20 years old and 795 individuals with pregnant or unmeasured blood pressure were excluded. We further excluded 3,241 individuals because the levels of serum aldehydes were not analyzed. As a result, a total of 1,733 individuals were included in the final analysis. The flowchart of participants selection was shown in Figure 1. The NHANES protocol was approved by the NCHS Institutional Review Committee. All the participants provided written informed consent to authorize the use of data.

### Study Outcome

Blood pressure (BP) was measured by trained investigators using a standardized protocol. Before BP measurements in the mobile examination center, all participants were asked to rest quietly in a seated position for 5 min. Three consecutive blood pressure readings were obtained when the participants maximum inflation level had been determined. The mean values of the three readings was used to calculate the average BP. Participants were diagnosed as having hypertension if the average SBP was ≥ 140 mm Hg, or the average DBP ≥ 90 mm Hg, or their answer to the question “Are you now taking prescribed medicine for high blood pressure?” was “yes”.

### Serum Aldehyde Measurement

Blood samples were collected in specially designed tubes and serum was separated using a microcentrifuge. Serum aldehydes were measured in a randomly selected one-third subsample of participants 12 years and older. Given that aldehydes tend to react with biological molecules to form various organic compounds including Schiff base protein adducts, free aldehydes that released into the headspace of biological samples from the Schiff base protein adducts at low pH (~3) were analyzed. The serum levels of aldehydes were measured *via* an automated analytical method using solid phase microextraction (SPME) gas chromatography (GC) and high-resolution mass spectrometry (HRMS). A detailed description regarding blood specimen sampling and detection procedures has been published elsewhere (22, 23). In human serum from NHANES, this analytical method quantitatively detects trace levels of 12 aldehydes (pentanaldehyde, propanaldehyde, octanaldehyde, o-toluenaldehyde, non-analdehyde, isopentanaldehyde, heptanaldehyde, hexanaldehyde, decanaldehyde, crotonaldehyde, benzaldehyde, and butyraldehyde). Among the group of 12 aldehydes, we selected 6 kinds of aldehydes which were detectable in more than 80% of NHANES participants, including benzaldehyde, butyraldehyde, heptanaldehyde, hexanaldehyde, isopentanaldehyde, and propanaldehyde. Values below the detection limit (LOD) of each aldehydes was replaced with the LOD divided by the square root of 2.

### Covariates

Covariates related to exposure or outcome were obtained and controlled in multivariate logistic regression. They were age, sex (male, female), race/ethnicity (non-Hispanic white, non-Hispanic black, Hispanic, non-Hispanic Asian, and other), body mass index (BMI), educational level (lower than high school, high school graduate or general equivalency diploma, some college or associate degree, and bachelor' s degree or above), self-reported history of cardiovascular diseases (congestive heart failure, coronary heart disease, angina/angina pectoris, heart attack, or stroke), diabetes status (self-reported diagnosis by a healthcare provider), smoking status (current, former, or never), alcohol use (yes or no), ratio of family income to poverty (≤1, 1–3, >3), and physical activity (inactive, insufficiently active, active). BMI was calculated as weight in kilograms divided by height in meters squared. Alcohol drinkers were defined as who consumed at least 12 drinks in the last 12 months. One drink was defined for NHANES participants as 12 ounces of beer, a 5-ounce glass of wine, or 1.5 ounces of liquor. Physical activity was classed to active group [defined as the product of the metabolic equivalent value (MET = 1 kcal/h per kilometer body weight) of 7.5 or more], insufficiently active group (defined as the product of the MET value ranging from 3.75 to 7.5), and inactive group (defined as the product of the MET value <3.75).

### Statistical Analysis

Continuous variables are summarized as the mean with standard deviation (SD), and categorical variables as the number with percentage. The serum levels of aldehydes were divided into quartiles using the lowest quartile as the reference group. Multivariate logistic regression models were adopted to evaluate the associations between aldehydes exposure and hypertension. Three models were developed to gradually adjust the confounders: model 1 was adjusted for age and sex; model 2 was further adjusted for education level, race, smoking, alcohol use, poverty income ratio, and physical activity; model 3 was further adjusted for BMI, diabetes, and cardiovascular disease. The shape of the concentration–response relationship between Aldehydes and hypertension were evaluated using generalized additive models through spline function.

Subgroup analyses were conducted to investigate whether these associations were modified by age, sex, education level, race, physical activity, alcohol use, smoking, diabetes, cardiovascular disease, and BMI. Additionally, two sensitivity analyses were conducted to further evaluate the robustness of study findings: (1) restricted to participants free of diabetes and cardiovascular disease; (2) used an alternative cut-off value of 130/80 mm Hg to define hypertension according to the 2017 AHA/ACA blood pressure guideline; (3) including participants aged 18–20 years old; (4) excluding participants reporting extreme total energy intakes (<850 or >4,000 kcal/day) (24) or currently breastfeeding a baby; (5) further adjusting for total energy intake and marital status. All statistical analyses were conducted using R version 4.0.2 (R Foundation for Statistical Computing, Vienna, Austria). A two-sided *P*-value of 0.05 or less was considered statistically significant.

## Results

### Population Characteristics

Table 1 showed the general characteristics of study population. The mean age was 48.0 ± 16.7 years and an approximately equivalent of sex distribution was noted (female 49.9%, male 50.1%). The Non-Hispanic White was the most common ethnic type (49.1%). The prevalence of hypertension in included participants was 18.3%. Compared to individuals without hypertension, those with hypertension seem to be older, more likely to be Hispanic, drinkers. Additionally, the trends of a higher proportion of cardiovascular disease, inactive exercise, higher level of BMI and hexanaldehyde were also observed in individuals with hypertension.

**Table 1:** Characteristics of participants in NHANES 2013–2014.

**Characteristics**	**Total**	**Non-hypertension**	**Hypertension**	***P***
	**(*n* = 1,733)**	**(*n* = 1,416)**	**(*n* = 317)**	
Age (years)	48.0 ± 16.7	45.2 ± 16.0	60.7 ± 13.3	<0.001
**Sex**				0.923
Female	865 (49.9%)	706 (49.9%)	159 (50.2%)	
Male	868 (50.1%)	710 (50.1%)	158 (49.8%)	
**Race**				<0.001
Hispanic	351 (20.3%)	303 (21.4%)	48 (15.1%)	
Non-Hispanic White	850 (49.1%)	703 (49.7%)	147 (46.4%)	
Non-Hispanic Black	318 (18.4%)	229 (16.2%)	89 (28.1%)	
Non-Hispanic Asian	155 (8.9%)	131 (9.3%)	24 (7.6%)	
Other Race—Including Multi-Racial	59 (3.4%)	50 (3.5%)	9 (2.8%)	
**Education level**				0.708
< High school	388 (22.4%)	309 (21.8%)	79 (24.9%)	
High school graduate or general equivalency diploma	408 (23.5%)	333 (23.5%)	75 (23.7%)	
Some college or associate degree	555 (32.0%)	455 (32.1%)	100 (31.6%)	
≥Bachelor' s degree	381 (22.0%)	318 (22.5%)	63 (19.9%)	
Unknown	1 (0.1%)	1 (0.1%)	0 (0%)	
**Drinking**				0.001
Yes	250 (14.4%)	183 (12.9%)	67 (21.1%)	
No	1,171 (67.6%)	979 (69.1%)	192 (60.6%)	
Missing	312 (18.0%)	254 (17.9%)	58 (18.3%)	
**Smoking**				0.235
Never	694 (40.1%)	577 (40.8%)	117 (36.9%)	
Former	309 (17.8%)	243 (17.2%)	66 (20.8%)	
Current	730 (42.1%)	596 (42.1%)	134 (42.3%)	
**Cardiovascular disease**				<0.001
No	1,559 (90.0%)	1,295 (91.5%)	264 (83.3%)	
Yes	174 (10.0%)	121 (8.6%)	53 (16.7%)	
**Diabetes**				0.153
No	1,501 (86.6%)	1,236 (87.3%)	265 (83.6%)	
Yes	183 (10.6%)	144 (10.2%)	39 (12.3%)	
Unknown	49 (2.8%)	36 (2.5%)	13 (4.1%)	
**Ratio of family income to poverty**				0.331
≤ 1	413 (23.8%)	343 (24.2%)	70 (22.1%)	
1–3	633 (36.5%)	510 (36.0%)	123 (38.8%)	
>3	578 (33.4%)	468 (33.1%)	110 (34.7%)	
Unknown	109 (6.3%)	95 (6.7%)	14 (4.4%)	
**Exercise**				0.001
Inactive	755 (43.6%)	591 (41.7%)	164 (51.7%)	
Insufficiently active	95 (5.5%)	72 (5.1%)	23 (7.3%)	
Active	881 (50.8%)	752 (53.1%)	129 (40.7%)	
Unknown	2 (0.1%)	1 (0.1%)	1 (0.3%)	
Body mass index (kg/m^2^)	28.8 ± 6.9	28.6 ± 6.8	29.8 ± 7.4	0.003
Benzaldehyde (ng/mL)	1.6 ± 1.8	1.6 ± 1.7	1.6 ± 2.4	0.570
Butyraldehyde (ng/mL)	0.6 ± 0.5	0.6 ± 0.3	0.6 ± 1.0	0.055
Heptanaldehyde (ng/mL)	0.5 ± 0.2	0.5 ± 0.2	0.5 ± 0.3	0.815
Hexanaldehyde (ng/mL)	2.3 ± 1.9	2.3 ± 1.1	2.6 ± 3.9	0.005
Isopentanaldehyde (ng/mL)	0.8 ± 0.6	0.8 ± 0.6	0.7 ± 0.5	0.092
Propanaldehyde (ng/mL)	2.2 ± 1.1	2.2 ± 1.0	2.3 ± 1.4	0.368

*Data are presented as mean with standard deviation, or number with percentage*.

*NHANES, National Health and Nutrition Examination Survey*.

### Associations Between Aldehydes and Hypertension

**Figure 2:**
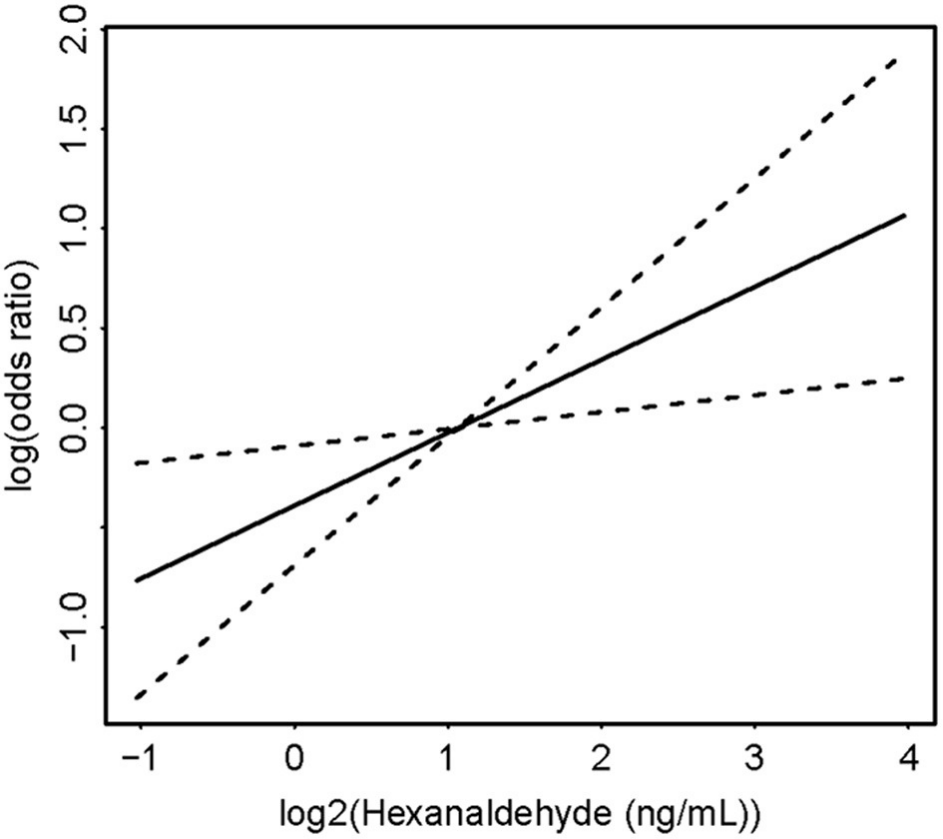
Concentration-response curve of association between hexanaldehyde and hypertension.

The serum concentrations of 6 selected aldehydes were shown in Table 1. There seems to be a numerically higher level of hexanaldehyde in participants with hypertension when compared to participants without hypertension (2.6 ± 3.9 vs. 2.3 ± 1.1 ng/mL). Table 2, Figure 2, and Supplementary Figure 1 summarized the associations of the quartiles of aldehydes with the odds of hypertension in three multivariate logistic regression models. After fully adjusting for demographic characteristics and other covariates (Model 3), the odds ratios (ORs) with 95% confidence intervals (CIs) for hypertension in participants from the highest quartile of serum aldehydes concentration were 0.77 (0.46–1.28), 1.35 (0.85–2.16), 1.07 (0.65–1.75), 2.15 (1.33–3.51), 1.09 (0.55–2.16), and 1.08 (0.65–1.79) for benzaldehyde, butyraldehyde, heptanaldehyde, hexanaldehyde, isopentanaldehyde, and propanaldehyde, respectively, in comparison to those with the lowest quartile. Among the 6 selected aldehydes, only serum hexanaldehyde was found to be significantly associated with hypertension in NHANES adults.

**Table 2:** Associations of selected aldehydes with odds of hypertension.

**Aldehydes**	**OR (95% CI)**				***P*_trend_**
	**Q1**	**Q2**	**Q3**	**Q4**	
**Benzaldehyde**					
Model 1	Ref	0.82 (0.55–1.22)	0.70 (0.47–1.06)	0.69 (0.45–1.03)	0.052
Model 2	Ref	0.85 (0.53–1.36)	0.78 (0.48–1.28)	0.76 (0.46–1.23)	0.242
Model 3	Ref	0.86 (0.53–1.39)	0.83 (0.50–1.37)	0.77 (0.46–1.28)	0.318
**Butyraldehyde**					
Model 1	Ref	1.19 (0.81–1.76)	1.39 (0.94–2.06)	1.40 (0.96–2.07)	0.061
Model 2	Ref	1.21 (0.77–1.88)	1.42 (0.90–2.23)	1.37 (0.87–2.16)	0.136
Model 3	Ref	1.13 (0.72–1.80)	1.44 (0.91–2.29)	1.35 (0.85–2.16)	0.134
**Heptanaldehyde**					
Model 1	Ref	1.04 (0.70–1.55)	1.21 (0.81–1.81)	0.94 (0.62–1.44)	0.956
Model 2	Ref	1.05 (0.66–1.68)	1.26 (0.79–2.00)	1.04 (0.65–1.68)	0.666
Model 3	Ref	1.06 (0.65–1.73)	1.26 (0.78–2.03)	1.07 (0.65–1.75)	0.613
**Hexanaldehyde**					
Model 1	Ref	1.37 (0.91–2.07)	1.14 (0.75–1.74)	1.77 (1.19–2.67)	0.018
Model 2	Ref	1.63 (1.01–2.62)	1.25 (0.77–2.03)	2.20 (1.38–3.53)	0.005
Model 3	Ref	1.69 (1.03–2.76)	1.20 (0.73–1.99)	2.15 (1.33–3.51)	0.011
**Isopentanaldehyde**					
Model 1	Ref	1.07 (0.73–1.58)	1.28 (0.87–1.87)	1.15 (0.77–1.73)	0.324
Model 2	Ref	1.13 (0.72–1.79)	1.35 (0.80–2.28)	0.92 (0.47–1.78)	0.925
Model 3	Ref	1.16 (0.73–1.88)	1.52 (0.88–2.63)	1.09 (0.55–2.16)	0.552
**Propanaldehyde**					
Model 1	Ref	0.83 (0.56–1.23)	1.24 (0.85–1.81)	1.11 (0.75–1.64)	0.260
Model 2	Ref	0.89 (0.56–1.40)	1.31 (0.83–2.08)	1.11 (0.68–1.82)	0.371
Model 3	Ref	0.87 (0.54–1.39)	1.37 (0.86–2.18)	1.08 (0.65–1.79)	0.216

*Model 1: adjusted for age and sex*.

*Model 2: further adjusted for education level, race, smoking, alcohol use, poverty income ratio, and physical activity*.

*Model 3: further adjusted for body mass index, diabetes, and cardiovascular disease*.

*OR, odd ratio; CI, confidence interval; Q, quartile*.

### Subgroup Analyses

In subgroup analyses stratified by age, sex, education level, race, physical activity, alcohol use, smoking, diabetes, cardiovascular disease, and BMI, no significant interactions for the association between serum hexanaldehyde and the odds of hypertension were observed (Table 3). The subgroup analyses of the remaining 5 aldehydes were depicted in Supplementary Tables 1–5.

**Table 3:** Stratified analyses between hexanaldehyde and odds of hypertension.

**Subgroups**	**OR (95% CI)**					***P*_trend_**	***P*_inter_**
	**Case/total**	**Q1**	**Q2**	**Q3**	**Q4**		
**Sex**							0.676
Female	159/865	Ref	2.12 (1.07–4.36)	1.43 (0.69–3.01)	1.94 (0.96–4.02)	0.597	
Male	158/868	Ref	1.26 (0.60–2.67)	1.17 (0.57–2.40)	2.12 (1.07–4.27)	0.033	
**Age (years)**							0.760
<65	178/1,397	Ref	1.34 (0.71–2.54)	0.97 (0.50–1.89)	2.22 (1.23–4.06)	0.020	
≥65	139/336	Ref	1.58 (0.67–3.78)	1.62 (0.70–3.80)	1.94 (0.82–4.71)	0.148	
**Education (years)**							0.725
<13	254/1,351	Ref	1.67 (0.95–2.95)	0.97 (0.54–1.76)	2.16 (1.25–3.78)	0.035	
≥13	63/381	Ref	1.20 (0.40–3.65)	1.63 (0.57–4.73)	1.42 (0.48–4.22)	0.424	
**Race**							0.418
Hispanic	48/351	Ref	2.02 (0.47–9.92)	3.13 (0.70–16.25)	2.55 (0.62–12.24)	0.215	
Non–Hispanic	269/1,382	Ref	1.40 (0.83–2.38)	1.14 (0.67–1.96)	1.85 (1.11–3.13)	0.048	
**Exercise**							0.930
Inactive/insufficiently active	187/850	Ref	1.72 (0.87–3.42)	1.28 (0.65–2.53)	2.11 (1.08–4.18)	0.072	
Active	129/881	Ref	1.21 (0.57–2.55)	1.09 (0.50–2.38)	2.06 (1.01–4.25)	0.063	
**Drinking**							0.512
Never	67/250	Ref	0.74 (0.24–2.29)	0.53 (0.16–1.70)	1.42 (0.48–4.33)	0.615	
Ever	192/1,171	Ref	1.78 (1.01–3.19)	1.49 (0.84–2.64)	2.27 (1.30–4.04)	0.012	
**Smoking**							0.242
Never	117/694	Ref	1.20 (0.52–2.83)	1.12 (0.47–2.69)	1.83 (0.82–4.19)	0.163	
Ever	200/1,039	Ref	1.96 (1.06–3.66)	1.23 (0.64–2.36)	2.62 (1.43–4.88)	0.012	
**Diabetes**							0.896
No	265/1,501	Ref	1.69 (1.00–2.88)	1.13 (0.66–1.95)	2.18 (1.30–3.68)	0.018	
Yes	39/183	Ref	4.03 (0.42–47.04)	1.26 (0.21–8.34)	0.95 (0.10–8.89)	0.842	
**Cardiovascular disease**							0.686
No	264/1,559	Ref	1.49 (0.88–2.54)	0.95 (0.55–1.65)	2.07 (1.24–3.50)	0.031	
Yes	53/174	Ref	1.15 (0.21–6.22)	4.90 (0.96–30.92)	1.77 (0.33–10.09)	0.252	
**Body mass index (kg/m** ^**2**^ **)**							0.295
<25	79/538	Ref	0.82 (0.26–2.55)	1.72 (0.60–5.04)	0.92 (0.29–2.94)	0.702	
≥25	236/1,183	Ref	1.74 (0.99–3.10)	0.95 (0.52–1.75)	2.49 (1.44–4.34)	0.010	

*Covariates adjusted in the model are age, sex, education level, race, smoking, alcohol use, poverty income ratio, physical activity, body mass index, diabetes mellitus, and cardiovascular disease, when they were not the strata variables*.

*OR, odd ratio; CI, confidence interval; Q, quartile*.

*P_trend_, P-values for trend analysis of the relationship between hexanaldehyde and hypertension*.

*P_inter_, P-values for interaction test*.

### Sensitivity Analyses

After excluding participants with diabetes or cardiovascular disease (*n* = 304), the positive association between hexanaldehyde and the odds of hypertension was still significant (Table 4). A similar result was also found when using an updated 130/80 mm Hg to define hypertension in included participants. Likewise, the results from sensitivity analyses of the remaining five aldehydes did not significantly change when compared to main analyses.

**Table 4:** Sensitivity analysis of associations between selected aldehydes and odds of hypertension.

**Aldehydes**	**OR (95% CI)**				***P*_trend_**
	**Q1**	**Q2**	**Q3**	**Q4**	
**Restricted to participants free of diabetes and cardiovascular disease (*****n*** **= 1,429)^a^**					
Benzaldehyde	Ref	0.99 (0.57–1.72)	0.80 (0.44–1.43)	0.85 (0.47–1.52)	0.478
Butyraldehyde	Ref	1.19 (0.70–2.03)	1.49 (0.87–2.54)	1.41 (0.82–2.44)	0.154
Heptanaldehyde	Ref	1.24 (0.70–2.20)	1.52 (0.86–2.70)	1.45 (0.81–2.59)	0.154
Hexanaldehyde	Ref	1.63 (0.94–2.83)	0.93 (0.51–1.68)	2.24 (1.30–3.91)	0.028
Isopentanaldehyde	Ref	1.07 (0.63–1.84)	1.15 (0.61–2.15)	1.21 (0.57–2.62)	0.608
Propanaldehyde	Ref	0.79 (0.45–1.38)	1.56 (0.92–2.67)	1.00 (0.55–1.82)	0.433
**Using 130/80 mm Hg to define hypertension (*****n*** **= 1,733)^b^**					
Benzaldehyde	Ref	0.87 (0.59–1.29)	0.78 (0.52–1.18)	0.66 (0.43–1.00)	0.044
Butyraldehyde	Ref	1.07 (0.74–1.55)	1.41 (0.97–2.06)	1.07 (0.73–1.57)	0.454
Heptanaldehyde	Ref	1.15 (0.77–1.70)	1.31 (0.88–1.94)	1.00 (0.67–1.48)	0.855
Hexanaldehyde	Ref	1.27 (0.86–1.89)	0.98 (0.66–1.45)	1.60 (1.08–2.38)	0.073
Isopentanaldehyde	Ref	0.86 (0.58–1.26)	1.05 (0.67–1.62)	0.67 (0.39–1.15)	0.253
Propanaldehyde	Ref	1.15 (0.78–1.69)	1.55 (1.06–2.28)	1.09 (0.72–1.65)	0.362
**Included participants aged 18–20 years old (*****n*** **= 1,830)**					
Benzaldehyde	Ref	1.12 (0.68–1.84)	1.31 (0.80–2.13)	1.14 (0.69–1.88)	0.508
Butyraldehyde	Ref	1.14 (0.72–1.82)	1.36 (0.85–2.18)	1.28 (0.80–2.05)	0.237
Heptanaldehyde	Ref	1.12 (0.68–1.84)	1.31 (0.80–2.13)	1.14 (0.69–1.88)	0.486
Hexanaldehyde	Ref	1.67 (1.02–2.76)	1.22 (0.73–2.04)	2.05 (1.25–3.38)	0.021
Isopentanaldehyde	Ref	1.08 (0.67–1.75)	1.32 (0.76–2.28)	0.94 (0.47–1.86)	0.904
Propanaldehyde	Ref	0.85 (0.52–1.38)	1.43 (0.89–2.30)	1.03 (0.62–1.73)	0.453
**Excluding participants reporting extreme total energy intakes or currently breastfeeding a baby (*****n*** **= 1,569)**					
Benzaldehyde	Ref	0.78 (0.46–1.32)	0.60 (0.34–1.05)	0.77 (0.45–1.33)	0.254
Butyraldehyde	Ref	1.23 (0.75–2.03)	1.55 (0.94–2.58)	1.59 (0.96–2.65)	0.049
Heptanaldehyde	Ref	1.27 (0.75–2.15)	1.17 (0.70–1.96)	1.06 (0.61–1.84)	0.870
Hexanaldehyde	Ref	1.53 (0.90–2.63)	1.34 (0.78–2.31)	2.23 (1.32–3.81)	0.007
Isopentanaldehyde	Ref	1.11 (0.67–1.84)	1.33 (0.75–2.38)	1.05 (0.50–2.20)	0.675
Propanaldehyde	Ref	0.90 (0.54–1.49)	1.39 (0.84–2.31)	1.15 (0.66–2.01)	0.330
**Further adjusting for total energy intake and marital status (*****n*** **= 1,733)**					
Benzaldehyde	Ref	0.77 (0.46–1.26)	0.77 (0.46–1.29)	0.75 (0.45–1.25)	0.295
Butyraldehyde	Ref	1.08 (0.67–1.72)	1.43 (0.90–2.30)	1.35 (0.84–2.18)	0.129
Heptanaldehyde	Ref	1.08 (0.65–1.78)	1.19 (0.73–1.95)	1.04 (0.62–1.72)	0.773
Hexanaldehyde	Ref	1.62 (0.98–2.71)	1.18 (0.70–1.98)	2.34 (1.43–3.88)	0.005
Isopentanaldehyde	Ref	1.13 (0.70–1.83)	1.38 (0.80–2.41)	1.04 (0.52–2.10)	0.675
Propanaldehyde	Ref	0.74 (0.45–1.21)	1.35 (0.84–2.16)	1.07 (0.64–1.79)	0.362

a *Adjusted for age, sex, education level, race, smoking, alcohol use, poverty income ratio, physical activity and body mass index*.

b *Adjusted for age, sex, education level, race, smoking, alcohol use, poverty income ratio, physical activity, body mass index, diabetes, and cardiovascular disease*.

*OR, odd ratio; CI, confidence interval; Q, quartile*.

## Discussion

To the best of our knowledge, this study is the first to investigate the associations between aldehyde exposure and the prevalence of hypertension in a selected, representative population. We found that increased serum hexanaldehyde level was positively associated with odds of hypertension in U.S. adults. We did not observe statistically significant associations between the other aldehydes (benzaldehyde, butyraldehyde, heptanaldehyde, isopentanaldehyde, and propanaldehyde,) and the prevalent hypertension.

As far as we know, there is no epidemiological study investigating the relationship between aldehyde exposure and risk of hypertension. Available evidences on this topic were based on few animal studies. Previous research reported that when aldehydes were administrated at low doses in spontaneously hypertensive rats, they could induce a dose-dependent pressor response by sympathomimetic effects though the release of norepinephrine from adrenergic neurons (25, 26). In addition, subsequent study also found that kidney aldehyde conjugates were significantly elevated in fructose induced hypertensive rats and spontaneously hypertensive rats (27, 28). More importantly, dietary supplement of methylglyoxal to Wistar-Kyoto rats has led to adverse renal vascular changes and hypertension (28). In the current study, we firstly observed that increased serum hexanaldehyde were significantly associated with higher odds of hypertension. As reported, hexanaldehyde is a kind of saturated aldehydes and a widespread exposure from tobacco smoke (29). Toxicology research suggested that hexanaldehyde exposure could cause nasal obstruction and headaches in humans (30). Moreover, elevated concentration of blood and exhaled hexanaldehyde has been noted in lung-cancer patients (31, 32). However, no mechanism research and population-based study have focused on the effects of hexanaldehyde exposure on cardiovascular diseases including hypertension. The health effect of hexanaldehyde and the reasons for differences of various aldehydes need further investigated.

There are limited studies investigating the health effect of aldehydes exposure, partly because the trait of volatile and high reactivity have resulted in difficulties in qualitative and quantitative determination of aldehydes *in vivo*. In recent years, some researchers have developed and validated a multistep procedure using SPME-GC-HRMS to detect the serum aldehydes concentrations (22). On the basis of above method, several cross-sectional studies have examined the potential relationship between serum aldehydes and cardiovascular diseases or obesity (20, 21, 33). Xu et al. showed that serum isopentanaldehyde concentrations were positively associated with the odds of cardiovascular disease (20). Given the differences in study outcomes, it is difficult to directly compare our results with the abovementioned findings. Even though, these insights collectively suggested that aldehyde exposure should be considered as an important environment contributor to cardiovascular diseases.

Although aldehydes have been suggested to have cytotoxic, mutagenic, genotoxic and carcinogenic effects (34), the possible mechanism underlying the association between serum aldehydes and hypertension were not well-defined. First, aldehyde-covalent modification of proteins and other biological macromolecules could be toxic and be mediators of inflammation and immune response which contributed to the pathogenesis of vascular diseases such as hypertension (35). Second, methylglyoxal has been reported to decrease serum nitric oxide levels in rats model (27). Excess reactive aldehydes may inhibit endogenous nitric oxide (NO) synthase *via* deactivation of endothelial proteins and impair NO-mediated endothelial function (36). Third, as aldehydes with the electrophilic nature prefer to react with the free amino and sulfhydryl groups of proteins, adducted aldehydes could bind to sufhydryl group of L-type Ca^2+^ channel proteins thus altering their functions. This may increase cytosolic free calcium levels and peripheral vascular resistance, and induce hypertension (37). Finally, oxidative stress has been confirmed to play a vital role in hypertension development by multiple animal models. Acrolein formed from lipid peroxidation or polyamine metabolism can induce and participate in oxidative stress which contributes to endothelial dysfunction (38). Increased methylglyoxal level has also been shown to cause oxidative stress in rat vascular smooth muscle cells (39).

Our study showed some advantages. First, the study was the first to report the association of aldehydes with hypertension using a nationally representative sample of U.S. adults, which facilitates the generalization of our findings. Second, the comprehensive data collection in the NHANES allowed us to adjust for a multitude of potential confounding factors. However, several limitations should also be acknowledged as well. First, due to the cross-sectional study design, causality relationship cannot be determined in this study. Second, the exposure of aldehydes can be endogenous and exogenous. Although we have observed a positive relationship between aldehydes and hypertension, it is difficult for us to evaluate whether the level of aldehydes in serum are exogenous or endogenous. Finally, the participants in the current study were adults from the U.S. Therefore, it should be cautious to generalize our results to other populations.

## Conclusion

In summary, increased serum hexanaldehyde level was positively associated with odds of hypertension in U.S. adults. Future studies are warranted to verify the association between hexanaldehyde exposure and hypertension and the underlying mechanism.

## Data Availability Statement

Publicly available datasets were analyzed in this study. This data can be found at: https://www.cdc.gov/nchs/nhanes/index.htm.

## Ethics Statement

The studies involving human participants were reviewed and approved by NCHS Institutional Review Committee. The patients/participants provided their written informed consent to participate in this study.

## Author Contributions

YB: conceptualization, methodology, and formal analysis. YZ: investigation, data curation, formal analysis, and writing—original draft. ML: data curation and writing—original draft. WF: investigation and writing—reviewing and editing. All authors contributed to the article and approved the submitted version.

## Conflict of Interest

The authors declare that the research was conducted in the absence of any commercial or financial relationships that could be construed as a potential conflict of interest.

## Publisher's Note

All claims expressed in this article are solely those of the authors and do not necessarily represent those of their affiliated organizations, or those of the publisher, the editors and the reviewers. Any product that may be evaluated in this article, or claim that may be made by its manufacturer, is not guaranteed or endorsed by the publisher.

## Footnotes

^1^CDC National Health and Nutrition Examination Survey. 2013–2014.
