# Supplementary material for: Association Between Serum Aldehydes and Hypertension in Adults: A Cross-Sectional Analysis of the National Health and Nutrition Examination Survey
Source: Front Cardiovasc Med. 2022 Mar 7;9:813244. doi: 10.3389/fcvm.2022.813244 (PMC8934859; doi:10.3389/fcvm.2022.813244)
Supplement: Supplementary file 1 [file Data_Sheet_1.docx]

**Supplementary Data**

**Table S1**

Stratified analyses between benzaldehyde and odds of hypertension.

| **Subgroups** | **OR (95% CI)** | | | | | ***P*_trend_** | ***P*_inter_** |
| --- | --- | --- | --- | --- | --- | --- | --- |
|  | **Case/total** | **Q1** | **Q2** | **Q3** | **Q4** |  |  |
| **Sex** |  |  |  |  |  |  | 0.328 |
| Female | 159/865 | Ref | 0.77 (0.39-1.55) | 0.87 (0.43-1.77) | 0.79 (0.37-1.64) | 0.621 |  |
| Male | 158/868 | Ref | 0.85 (0.41-1.76) | 0.64 (0.30-1.36) | 0.59 (0.28-1.23) | 0.171 |  |
| **Age (years)** |  |  |  |  |  |  | 0.184 |
| < 65 | 178/1397 | Ref | 1.01 (0.56-1.80) | 0.97 (0.52-1.80) | 0.52 (0.26-1.01) | 0.075 |  |
| ≥ 65 | 139/336 | Ref | 0.95 (0.37-2.47) | 0.64 (0.25-1.63) | 2.33 (0.92-6.09) | 0.166 |  |
| **Education (years)** |  |  |  |  |  |  | 0.554 |
| < 13 | 254/1351 | Ref | 0.81 (0.46-1.42) | 0.78 (0.45-1.37) | 0.68 (0.38-1.22) | 0.204 |  |
| ≥ 13 | 63/381 | Ref | 2.21 (0.74-6.92) | 0.94 (0.27-3.17) | 1.11 (0.35-3.52) | 0.761 |  |
| **Race** |  |  |  |  |  |  | 0.543 |
| Hispanic | 48/351 | Ref | 0.64 (0.17-2.31) | 0.59 (0.13-2.39) | 0.61 (0.16-2.26) | 0.476 |  |
| Non-Hispanic | 269/1382 | Ref | 1.08 (0.63-1.83) | 0.78 (0.44-1.34) | 0.90 (0.52-1.57) | 0.489 |  |
| **Exercise** |  |  |  |  |  |  | 0.892 |
| Inactive/ Insufficiently active | 187/850 | Ref | 1.40 (0.72-2.73) | 0.94 (0.47-1.87) | 0.88 (0.44-1.75) | 0.470 |  |
| Active | 129/881 | Ref | 0.76 (0.33-1.70) | 1.06 (0.49-2.30) | 0.75 (0.33-1.71) | 0.697 |  |
| **Drinking** |  |  |  |  |  |  | 0.839 |
| Never | 67/250 | Ref | 1.75 (0.52-6.17) | 0.59 (0.16-2.06) | 1.24 (0.36-4.42) | 0.752 |  |
| Ever | 192/1171 | Ref | 0.90 (0.51-1.56) | 0.95 (0.54-1.66) | 0.75 (0.42-1.35) | 0.401 |  |
| **Smoking** |  |  |  |  |  |  | 0.258 |
| Never | 117/694 | Ref | 1.45 (0.63-3.38) | 0.86 (0.36-2.05) | 0.95 (0.39-2.30) | 0.594 |  |
| Ever | 200/1039 | Ref | 1.10 (0.59-2.07) | 1.24 (0.66-2.33) | 0.96 (0.51-1.80) | 0.999 |  |
| **Body mass index (kg/m^2^)** |  |  |  |  |  |  | 0.147 |
| < 25 | 79/538 | Ref | 1.85 (0.68-5.14) | 0.47 (0.14-1.48) | 0.49 (0.13-1.66) | 0.091 |  |
| ≥ 25 | 236/1183 | Ref | 0.88 (0.48-1.58) | 0.95 (0.52-1.70) | 1.06 (0.59-1.90) | 0.793 |  |

OR, odd ratio. CI, confidence interval. Q, quartile.

*P*_trend_: *P* values for trend analysis of the relationship between benzaldehyde and hypertension

*P*_inter_: *P* values for interaction test

**Table S2**

Stratified analyses between butyraldehyde and odds of hypertension.

| **Subgroups** | **OR (95% CI)** | | | | | ***P*_trend_** | ***P*_inter_** |
| --- | --- | --- | --- | --- | --- | --- | --- |
|  | **Case/total** | **Q1** | **Q2** | **Q3** | **Q4** |  |  |
| **Sex** |  |  |  |  |  |  | 0.430 |
| Female | 159/865 | Ref | 0.89 (0.47-1.70) | 1.05 (0.54-2.01) | 1.26 (0.68-2.36) | 0.613 |  |
| Male | 158/868 | Ref | 1.98 (0.97-4.11) | 2.03 (0.99-4.26) | 2.02 (0.96-4.32) | 0.090 |  |
| **Age (years)** |  |  |  |  |  |  | 0.097 |
| < 65 | 178/1397 | Ref | 0.87 (0.49-1.57) | 1.13 (0.63-2.05) | 1.18 (0.66-2.10) | 0.423 |  |
| ≥ 65 | 139/336 | Ref | 1.50 (0.66-3.45) | 2.31 (1.03-5.27) | 1.58 (0.68-3.70) | 0.155 |  |
| **Education (years)** |  |  |  |  |  |  | 0.724 |
| < 13 | 254/1351 | Ref | 1.02 (0.59-1.76) | 1.47 (0.87-2.50) | 1.41 (0.83-2.39) | 0.111 |  |
| ≥ 13 | 63/381 | Ref | 1.42 (0.53-3.95) | 1.65 (0.59-4.75) | 1.34 (0.46-4.04) | 0.543 |  |
| **Race** |  |  |  |  |  |  | 0.682 |
| Hispanic | 48/351 | Ref | 1.71 (0.51-6.07) | 2.72 (0.78-10.29) | 1.38 (0.37-5.28) | 0.503 |  |
| Non-Hispanic | 269/1382 | Ref | 0.95 (0.58-1.57) | 1.16 (0.70-1.91) | 1.28 (0.78-2.11) | 0.251 |  |
| **Exercise** |  |  |  |  |  |  | 0.844 |
| Inactive/ Insufficiently active | 187/850 | Ref | 1.12 (0.60-2.11) | 1.52 (0.81-2.87) | 1.27 (0.68-2.40) | 0.332 |  |
| Active | 129/881 | Ref | 1.54 (0.75-3.18) | 1.58 (0.76-3.30) | 1.67 (0.81-3.46) | 0.183 |  |
| **Drinking** |  |  |  |  |  |  | 0.812 |
| Never | 67/250 | Ref | 0.82 (0.29-2.27) | 0.60 (0.20-1.72) | 0.92 (0.34-2.49) | 0.760 |  |
| Ever | 192/1171 | Ref | 1.12 (0.65-1.92) | 1.83 (1.09-3.09) | 1.33 (0.78-2.29) | 0.123 |  |
| **Smoking** |  |  |  |  |  |  | 0.899 |
| Never | 117/694 | Ref | 1.43 (0.67-3.10) | 1.44 (0.66-3.14) | 1.34 (0.64-2.89) | 0.465 |  |
| Ever | 200/1039 | Ref | 1.07 (0.59-1.92) | 1.23 (0.69-2.18) | 1.29 (0.72-2.31) | 0.340 |  |
| **Body mass index (kg/m^2^)** |  |  |  |  |  |  | 0.639 |
| < 25 | 79/538 | Ref | 2.20 (0.75-6.68) | 2.19 (0.77-6.50) | 1.75 (0.60-5.31) | 0.337 |  |
| ≥ 25 | 236/1183 | Ref | 0.97 (0.57-1.64) | 1.11 (0.65-1.89) | 1.22 (0.72-2.08) | 0.400 |  |

OR, odd ratio. CI, confidence interval. Q, quartile.

*P*_trend_: *P* values for trend analysis of the relationship between butyraldehyde and hypertension

*P*_inter_: *P* values for interaction test

**Table S3**

Stratified analyses between heptanaldehyde and odds of hypertension.

| **Subgroups** | **OR (95% CI)** | | | | | ***P*_trend_** | ***P*_inter_** |
| --- | --- | --- | --- | --- | --- | --- | --- |
|  | **Case/total** | **Q1** | **Q2** | **Q3** | **Q4** |  |  |
| **Sex** |  |  |  |  |  |  | 0.752 |
| Female | 159/865 | Ref | 1.06 (0.54-2.11) | 1.35 (0.68-2.69) | 1.17 (0.57-2.37) | 0.750 |  |
| Male | 158/868 | Ref | 0.98 (0.47-2.04) | 1.34 (0.64-2.81) | 1.16 (0.56-2.42) | 0.747 |  |
| **Age (years)** |  |  |  |  |  |  | 0.248 |
| < 65 | 178/1397 | Ref | 0.93 (0.50-1.70) | 1.23 (0.69-2.22) | 0.96 (0.52-1.76) | 0.860 |  |
| ≥ 65 | 139/336 | Ref | 1.75 (0.70-4.44) | 1.48 (0.58-3.79) | 1.35 (0.55-3.31) | 0.599 |  |
| **Education (years)** |  |  |  |  |  |  | 0.876 |
| < 13 | 254/1351 | Ref | 1.13 (0.64-1.98) | 1.24 (0.71-2.16) | 1.18 (0.67-2.09) | 0.507 |  |
| ≥ 13 | 63/381 | Ref | 1.39 (0.47-4.14) | 2.30 (0.78-7.07) | 1.19 (0.40-3.55) | 0.541 |  |
| **Race** |  |  |  |  |  |  | 0.159 |
| Hispanic | 48/351 | Ref | 1.09 (0.29-4.19) | 0.97 (0.25-3.83) | 0.33 (0.06-1.49) | 0.156 |  |
| Non-Hispanic | 269/1382 | Ref | 0.88 (0.51-1.48) | 1.22 (0.72-2.07) | 1.13 (0.67-1.93) | 0.423 |  |
| **Exercise** |  |  |  |  |  |  | 0.452 |
| Inactive/ Insufficiently active | 187/850 | Ref | 1.15 (0.61-2.17) | 0.87 (0.45-1.69) | 0.94 (0.50-1.77) | 0.688 |  |
| Active | 129/881 | Ref | 1.13 (0.51-2.53) | 1.69 (0.80-3.66) | 1.39 (0.62-3.18) | 0.255 |  |
| **Drinking** |  |  |  |  |  |  | 0.237 |
| Never | 67/250 | Ref | 0.87 (0.29-2.56) | 0.60 (0.19-1.83) | 0.56 (0.18-1.70) | 0.252 |  |
| Ever | 192/1171 | Ref | 1.18 (0.68-2.06) | 1.42 (0.81-2.49) | 1.30 (0.73-2.32) | 0.285 |  |
| **Smoking** |  |  |  |  |  |  | 0.122 |
| Never | 117/694 | Ref | 1.31 (0.56-3.09) | 1.86 (0.81-4.36) | 2.35 (1.05-5.44) | 0.028 |  |
| Ever | 200/1039 | Ref | 0.86 (0.47-1.60) | 1.17 (0.64-2.13) | 0.65 (0.33-1.25) | 0.412 |  |
| **Body mass index (kg/m^2^)** |  |  |  |  |  |  | 0.365 |
| < 25 | 79/538 | Ref | 0.41 (0.12-1.31) | 0.89 (0.28-2.80) | 1.71 (0.61-4.94) | 0.148 |  |
| ≥ 25 | 236/1183 | Ref | 1.27 (0.73-2.21) | 1.13 (0.64-1.98) | 0.92 (0.51-1.65) | 0.730 |  |

OR, odd ratio. CI, confidence interval. Q, quartile.

*P*_trend_: *P* values for trend analysis of the relationship between heptanaldehyde and hypertension

*P*_inter_: *P* values for interaction test

**Table S4**

Stratified analyses between isopentanaldehyde and odds of hypertension.

| **Subgroups** | **OR (95% CI)** | | | | | ***P*_trend_** | ***P*_inter_** |
| --- | --- | --- | --- | --- | --- | --- | --- |
|  | **Case/total** | **Q1** | **Q2** | **Q3** | **Q4** |  |  |
| **Sex** |  |  |  |  |  |  | 0.631 |
| Female | 159/865 | Ref | 0.72 (0.37-1.39) | 1.50 (0.72-3.11) | 1.30 (0.50-3.39) | 0.846 |  |
| Male | 158/868 | Ref | 0.97 (0.47-2.05) | 1.11 (0.49-2.52) | 0.74 (0.27-2.07) | 0.338 |  |
| **Age (years)** |  |  |  |  |  |  | 0.004 |
| < 65 | 178/1397 | Ref | 0.87 (0.48-1.56) | 1.15 (0.57-2.30) | 0.82 (0.35-1.91) | 0.742 |  |
| ≥ 65 | 139/336 | Ref | 1.97 (0.83-4.81) | 3.16 (1.29-8.09) | 2.47 (0.82-7.61) | 0.040 |  |
| **Education (years)** |  |  |  |  |  |  | 0.110 |
| < 13 | 254/1351 | Ref | 1.27 (0.75-2.19) | 1.36 (0.68-2.70) | 1.10 (0.50-2.44) | 0.761 |  |
| ≥ 13 | 63/381 | Ref | 0.55 (0.18-1.62) | 0.80 (0.29-2.17) | 1.34 (0.43-4.14) | 0.567 |  |
| **Race** |  |  |  |  |  |  | 0.290 |
| Hispanic | 48/351 | Ref | 0.26 (0.06-1.05) | 0.65 (0.18-2.28) | 0.38 (0.07-1.97) | 0.487 |  |
| Non-Hispanic | 269/1382 | Ref | 1.34 (0.81-2.26) | 1.46 (0.79-2.71) | 1.03 (0.49-2.20) | 0.750 |  |
| **Exercise** |  |  |  |  |  |  | 0.422 |
| Inactive/ Insufficiently active | 187/850 | Ref | 1.53 (0.80-2.98) | 3.48 (1.54-8.03) | 2.09 (0.81-5.49) | 0.104 |  |
| Active | 129/881 | Ref | 0.96 (0.47-1.98) | 0.84 (0.38-1.82) | 0.62 (0.22-1.77) | 0.419 |  |
| **Drinking** |  |  |  |  |  |  | 0.247 |
| Never | 67/250 | Ref | 0.74 (0.27-2.04) | 2.22 (0.58-8.63) | 1.07 (0.23-4.86) | 0.934 |  |
| Ever | 192/1171 | Ref | 0.94 (0.56-1.59) | 1.26 (0.69-2.31) | 1.00 (0.47-2.15) | 0.827 |  |
| **Smoking** |  |  |  |  |  |  | 0.186 |
| Never | 117/694 | Ref | 1.07 (0.50-2.32) | 0.97 (0.45-2.13) | 0.65 (0.27-1.54) | 0.334 |  |
| Ever | 200/1039 | Ref | 1.08 (0.58-2.00) | 0.98 (0.54-1.80) | 1.09 (0.60-2.01) | 0.856 |  |
| **Body mass index (kg/m^2^)** |  |  |  |  |  |  | 0.482 |
| < 25 | 79/538 | Ref | 0.84 (0.27-2.65) | 0.71 (0.17-3.09) | 1.28 (0.26-6.65) | 0.725 |  |
| ≥ 25 | 236/1183 | Ref | 1.23 (0.71-2.21) | 1.49 (0.82-2.73) | 1.08 (0.50-2.37) | 0.548 |  |

OR, odd ratio. CI, confidence interval. Q, quartile.

*P*_trend_: *P* values for trend analysis of the relationship between isopentanaldehyde and hypertension

*P*_inter_: *P* values for interaction test

**Table S5**

Stratified analyses between propanaldehyde and odds of hypertension.

| **Subgroups** | **OR (95% CI)** | | | | | ***P*_trend_** | ***P*_inter_** |
| --- | --- | --- | --- | --- | --- | --- | --- |
|  | **Case/total** | **Q1** | **Q2** | **Q3** | **Q4** |  |  |
| **Sex** |  |  |  |  |  |  | 0.475 |
| Female | 159/865 | Ref | 1.00 (0.53-1.89) | 1.23 (0.65-2.35) | 1.18 (0.59-2.36) | 0.845 |  |
| Male | 158/868 | Ref | 1.37 (0.66-2.90) | 1.56 (0.73-3.37) | 1.48 (0.67-3.29) | 0.116 |  |
| **Age (years)** |  |  |  |  |  |  | 0.011 |
| < 65 | 178/1397 | Ref | 0.71 (0.39-1.28) | 1.06 (0.60-1.87) | 0.76 (0.40-1.43) | 0.693 |  |
| ≥ 65 | 139/336 | Ref | 1.57 (0.66-3.79) | 3.70 (1.47-9.73) | 1.82 (0.72-4.67) | 0.086 |  |
| **Education (years)** |  |  |  |  |  |  | 0.567 |
| < 13 | 254/1351 | Ref | 1.25 (0.72-2.18) | 1.53 (0.88-2.67) | 1.48 (0.81-2.71) | 0.148 |  |
| ≥ 13 | 63/381 | Ref | 1.18 (0.45-3.12) | 0.53 (0.17-1.56) | 0.76 (0.28-2.03) | 0.386 |  |
| **Race** |  |  |  |  |  |  | 0.664 |
| Hispanic | 48/351 | Ref | 1.22 (0.34-4.44) | 1.95 (0.58-6.94) | 0.93 (0.25-3.49) | 0.869 |  |
| Non-Hispanic | 269/1382 | Ref | 0.86 (0.51-1.42) | 1.16 (0.71-1.92) | 1.05 (0.60-1.86) | 0.607 |  |
| **Exercise** |  |  |  |  |  |  | 0.698 |
| Inactive/ Insufficiently active | 187/850 | Ref | 0.87 (0.46-1.67) | 1.74 (0.93-3.30) | 1.08 (0.55-2.13) | 0.404 |  |
| Active | 129/881 | Ref | 0.90 (0.44-1.82) | 1.08 (0.53-2.21) | 1.00 (0.44-2.25) | 0.891 |  |
| **Drinking** |  |  |  |  |  |  | 0.917 |
| Never | 67/250 | Ref | 0.47 (0.15-1.33) | 1.31 (0.64-4.68) | 0.33 (0.09-1.10) | 0.428 |  |
| Ever | 192/1171 | Ref | 0.94 (0.55-1.61) | 1.21 (0.70-2.06) | 1.21 (0.67-2.17) | 0.397 |  |
| **Smoking** |  |  |  |  |  |  | 0.735 |
| Never | 117/694 | Ref | 1.00 (0.45-2.22) | 1.18 (0.56-2.51) | 0.96 (0.43-2.13) | 0.946 |  |
| Ever | 200/1039 | Ref | 1.20 (0.68-2.15) | 1.02 (0.55-1.89) | 1.26 (0.70-2.28) | 0.566 |  |
| **Body mass index (kg/m^2^)** |  |  |  |  |  |  | 0.581 |
| < 25 | 79/538 | Ref | 0.69 (0.21-2.20) | 1.24 (0.42-3.73) | 0.93 (0.27-3.28) | 0.827 |  |
| ≥ 25 | 236/1183 | Ref | 0.96 (0.56-1.63) | 1.39 (0.81-2.38) | 0.99 (0.55-1.78) | 0.673 |  |

OR, odd ratio. CI, confidence interval. Q, quartile.

*P*_trend_: *P* values for trend analysis of the relationship between propanaldehyde and hypertension

*P*_inter_: *P* values for interaction test


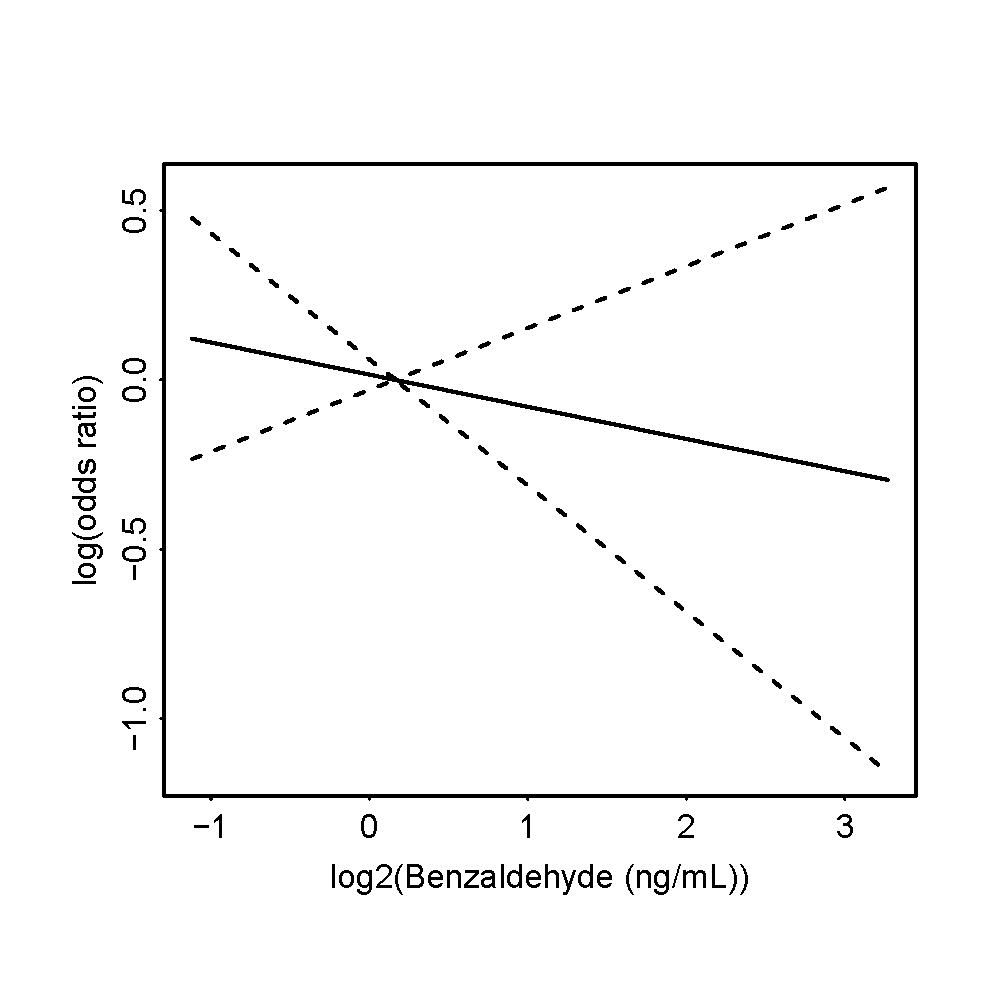

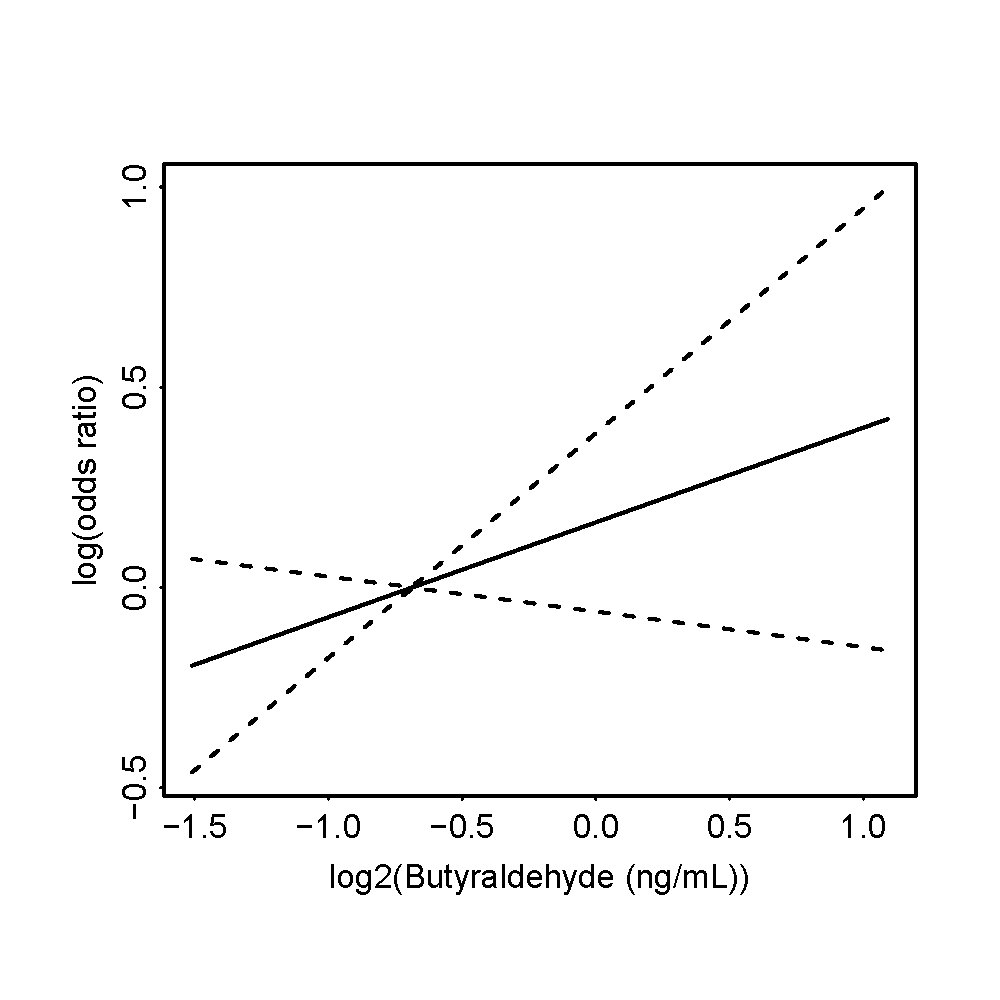


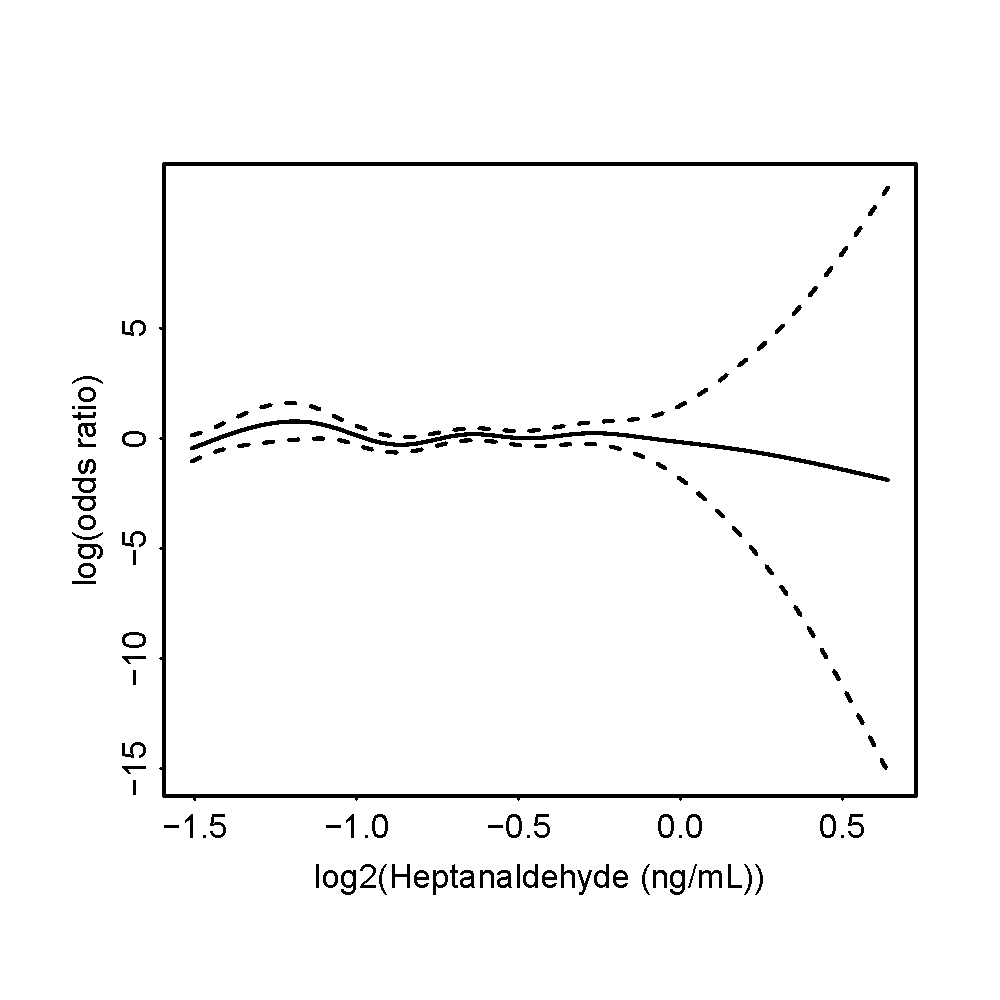

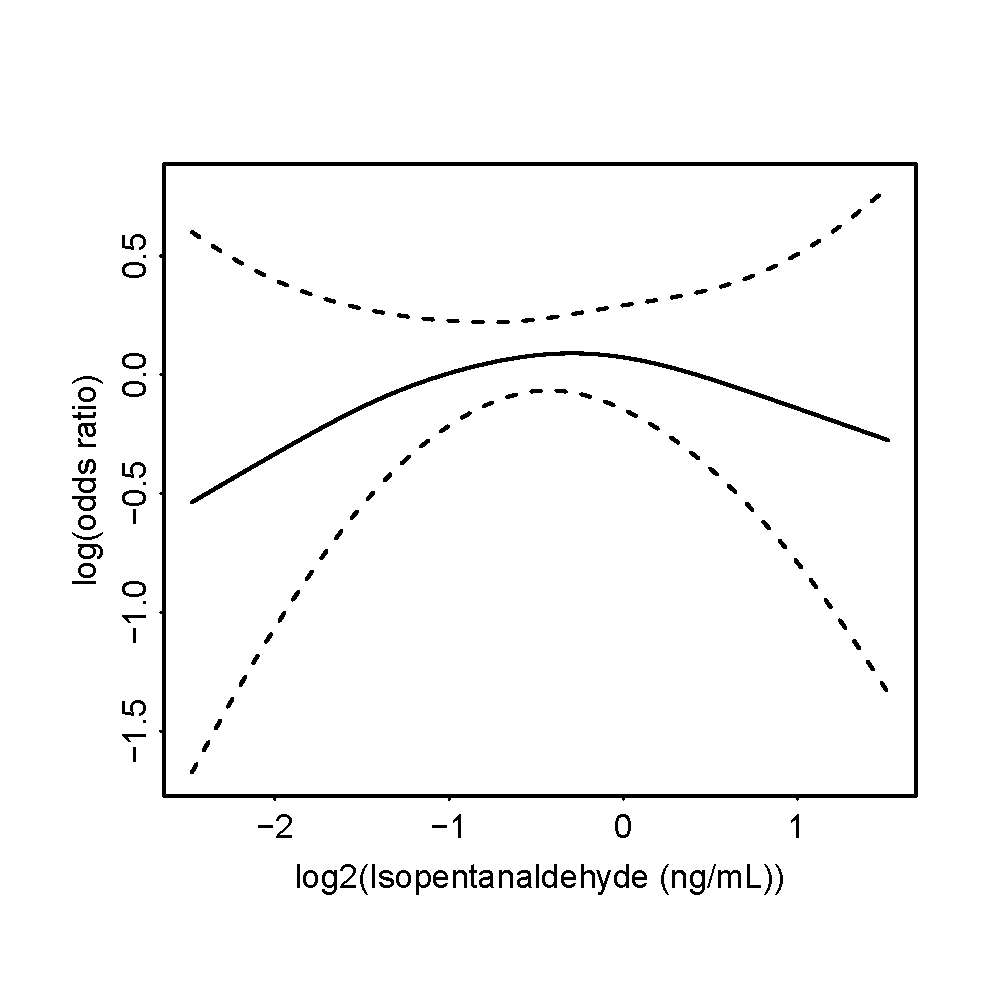


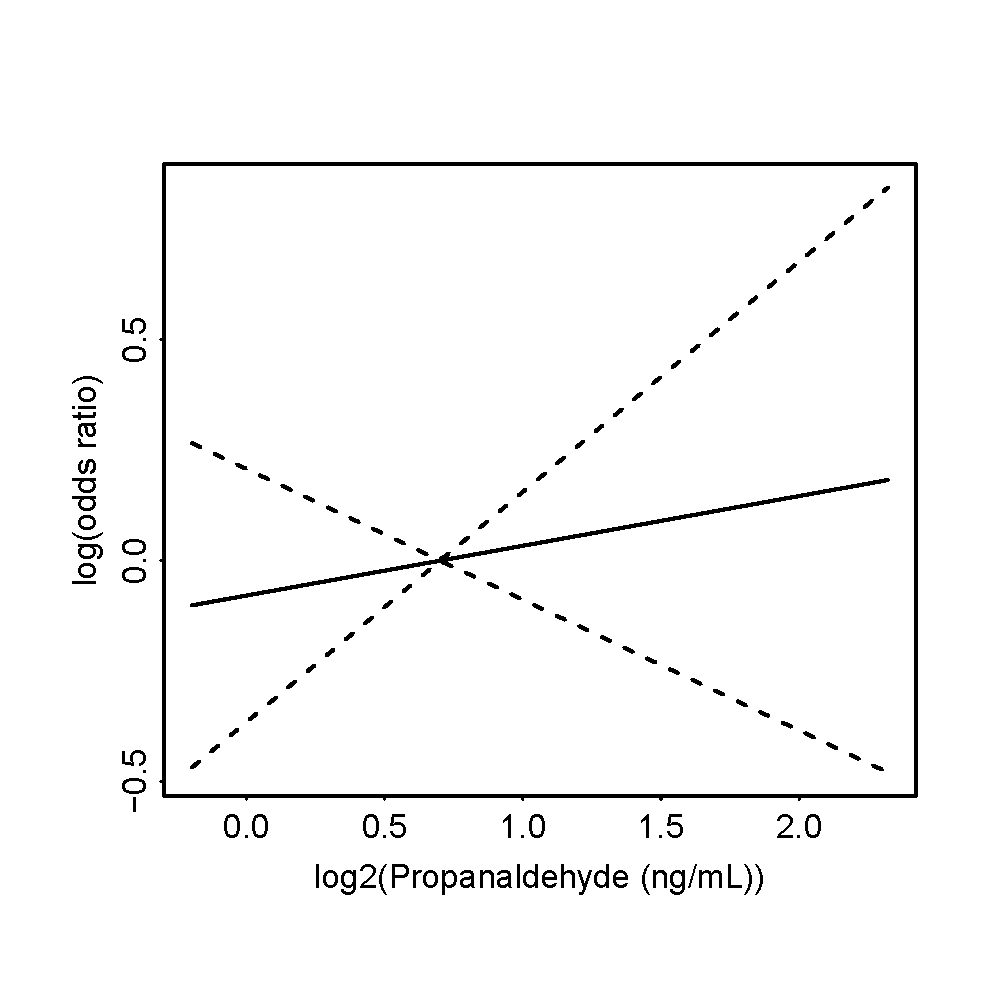


Figure S1. Concentration-response curve of association between benzaldehyde, butyraldehyde, heptanaldehyde, isopentanaldehyde, and propanaldehyde and hypertension.
